# Cisplatin increases carboxylesterases through increasing PXR mediated by the decrease of DEC1

**DOI:** 10.7555/JBR.37.20230047

**Published:** 2023-11-15

**Authors:** Minqin Xu, Lihua Zhang, Lan Lin, Zhiyi Qiang, Wei Liu, Jian Yang

**Affiliations:** Department of Pharmacology, Nanjing Medical University, Nanjing, Jiangsu 211166, China

**Keywords:** *cis*-diamminedichloroplatinum, pregnane X receptor, differentiated embryonic chondrocyte-expressed gene 1, carboxylesterase 1, carboxylesterase 2, irinotecan

## Abstract

*cis*-Diamminedichloroplatinum (CDDP) is widely used for the treatment of various solid cancers. Here we reported that CDDP increased the expression and enzymatic activities of carboxylesterase 1 (CES1) and carboxylesterase 2 (CES2), along with the upregulation of pregnane X receptor (PXR) and the downregulation of differentiated embryonic chondrocyte-expressed gene 1 (DEC1) in human hepatoma cells, primary mouse hepatocytes, mouse liver and intestine. The overexpression or knockdown of PXR alone upregulated or downregulated the CES1 and CES2 expression, respectively. The increases in CES1 and CES2 expression levels induced by CDDP abolished or enhanced by PXR knockdown or overexpression, implying that CDDP induces carboxylesterases through the activation of PXR. Likewise, the overexpression or knockdown of DEC1 alone significantly decreased or increased PXR and its targets. Moreover, the increases of PXR and its targets induced by CDDP were abolished or alleviated by the overexpression or knockdown of DEC1. The overexpression or knockdown of DEC1 affected the response of PXR to CDDP, but not vice versa, suggesting that CDDP increases carboxylesterases by upregulating PXR mediated by the decrease of DEC1. In addition, CDDP did not increase *DEC1* mRNA degradation but suppressed DEC1 promoter reporter activity, indicating that it suppresses DEC1 transcriptionally. The combined use of CDDP and irinotecan had a synergistic effect on two cell lines, especially when CDDP was used first.

## Introduction

Cisplatin or *cis*-diamminedichloroplatinum (CDDP) is one of the most effective and pioneering metal-based chemotherapeutic drugs ^[ 1] ^. It is widely used for the treatment of various solid cancers, such as cancers of head and neck, bladder, lung, cervical, colon and others ^[ 1– 3] ^. Besides its strong antitumor activity and long-lasting efficacy, CDDP has some side effects, such as drug resistance and serious adverse reactions including nausea, vomiting, nephrotoxicity, hepatotoxicity, ototoxicity, and neurotoxicity ^[ 4– 6] ^. The CDDP-induced toxic effects mentioned above are correlated with cytochrome P450 (P450 or CYP), such as CYP2E1 and CYP3A4/5 that produce reactive oxygen species ^[ 7] ^. In addition, CDDP can increase a systemic clearance, resulting in a reduced efficacy and even drug resistance due to the induction of its drug metabolizing enzymes (DMEs), such as CYP450 ^[ 8] ^. Therefore, the efficacy and toxicity of CDDP are closely related to DMEs.

The liver and intestine are among the primary sources of DMEs and play a determinant role in drug metabolism ^[ 9] ^. The transactivation by two major xenobiotic nuclear receptors, the pregnane X receptor (PXR) and the constitutive androstane receptor (CAR), is mainly responsible for the increased expression of these genes ^[ 10] ^. Carboxylesterases constitute a group of enzymes that catalyze the hydrolysis of drugs containing functional groups, such as carboxylic acid ester, amide, and thioester ^[ 10– 11] ^. The liver strongly expresses two major carboxylesterases, including human carboxylesterase 1 (CES1) and human carboxylesterase 2 (CES2), whereas the gastrointestinal tract mainly expresses CES2 ^[ 10, 12] ^. In addition to the difference in tissue distribution, the two enzymes hydrolyze distinct drugs. For instance, CES1 rapidly hydrolyzes clopidogrel, an antithrombogenic agent ^[ 12] ^; while CES2 rapidly hydrolyzes irinotecan (CPT11), an antitumor drug ^[ 12] ^. CDDP can increase CYP3A4 and p-gp through activating PXR ^[ 13– 14] ^. However, the effect of CDDP on other drug-metabolizing enzymes, such as carboxylesterases, remains uncertain.

Human differentiated embryonic chondrocyte-expressed gene 1 (DEC1), also named mouse stimulated with retinoic acid 13 (STRA13), and rat split and hairy-related protein (SHARP), is one of the basic helix-loop-helix transcriptional factors. It is correlated with cell differentiation, proliferation, biological rhythm and homeostasis of metabolism ^[ 15– 16] ^. DEC1 can be combined with retinoid X receptor alpha (RXRα), which impedes the formation of heterodimer of RXR and other nuclear receptors, such as PXR that leads to the repression of PXR function ^[ 17] ^. In addition, DEC1 represses CYP3A4 expression directly through binding to proximal promoter of CYP3A4 ^[ 18] ^.

In the present study, we found that CDDP increased CES1 and CES2 expressions accompanied by increasing PXR but decreasing DEC1 expression. It was hypothesized that CDDP increased CES1 and CES2 expressions and enzymatic activities through increasing PXR expression mediated by the decrease of DEC1 expression *in vitro* and *in vivo*. According to the fact that hydrolysis of CPT11 is required for its cytotoxicity, which is activated by hydrolyzed by CES2 ^[ 12] ^, it was assumed that the concurrent use of CDDP and CPT11 would enhance the activation of CPT11, thus making a synergistic effect. The outcome could contribute to guiding clinical rational combination of CDDP with CPT11 for the patients who suffered solid cancers, and is worth further studying and clinical trials.

## Materials and methods

### Reagents and materials

CDDP (Cat. #PHR1624), p-nitrophenylacetic acid (PNPA; Cat. #N20204-100G), clopidogrel hydrogen sulfate (Cat. #Y0001333), irinotecan hydrochloride (Cat. #I1406), rifampicin (RIF; Cat. #557303), and phenobarbital (Cat. #PHR8843) were purchased from Sigma-Aldrich (St. Louis, MO, USA). Dulbecco's modified Eagle medium (DMEM; Cat. #12430054) and TRIzol Reagent (Cat. #15596018CN) were from Invitrogen (Carlsbad, CA, USA). Trypase was from NCM Biotech (Cat. #C100C1, Suzhou, China). Monloney murine leukemia (MLV) reverse transcriptase (Cat. #M3681), RNase inhibitor (Cat. #N2111) and Luciferase Assay Reagent (Cat. #E1910) were purchased from Promega (Madison, WI, USA). Fetal bovine serum (FBS) was from Hyclone Laboratories (Cat. #SH30084.03, Logan, Utah, USA). The anti-CES1 was from Abcam (Cat. #ab45957, Cambridge, UK). The anti-CES2, anti-mPXR, and anti-CYP3A11 were kindly provided by Dr. Bingfang Yan (University of Rhode Island, Kingston, RI, USA). *PXR* shRNA and packaged lentiviral vectors containing *DEC1* shRNA (three viral strains) or lentiviral vectors (as control, shRNA) were from Genechem (Shanghai, China). The reporter constructs (pGL3-DEC1-1.3kb-Luc, pGL3-DEC1-1.1kb-Luc), DEC1 expressed construct and PXR expressed construct were kindly provided by Dr. Bingfang Yan. Restore PLUS Western Blot Stripping Buffer was from Thermo Fisher Scientific (Waltham, MA, USA). Antibody against DEC1 was from Santa Cruz (Cat. #sc-101023, Santa Cruz, CA, USA), antibody against β-actin was from Bioworld (Cat. #BS6007M, St. Louis Park, USA), and antibodies against CYP3A4 and PXR were from Abcam (Cat. #ab3572 and #ab118366, respectively). Goat anti-rabbit or anti-mouse IgG conjugated to horseradish peroxidase was from Proteintech Group (Cat. #SA00001-1, Chicago, Illinois, USA), and BCA protein assay kit were from Pierce Chemical (Cat. #23227, Pierce, Rockford, IL, USA). ECL Western blotting detection system was from Vazyme Biotech Co., Ltd. (Cat. #E412-02, Nanjing, Jiangsu, China). GenJet DNA Vitro Transfection Reagent (Ver. Ⅱ) was from SignaGen Laboratories (Cat. #SL100488, Gaithersburg, MD, USA). All other chemicals were obtained from Sigma-Aldrich.

### Cell culture and treatment

Hepatoma (HepG2) and colon adenocarcinoma (SW480) cells were from American Type Culture Collection (Manassas, VA, USA). Cells were seeded at the density of 5 × 10 ^6^ cells/well (6-well plates for protein level), 5 × 10 ^5^ cells/well (12-well plates for mRNA level), or 5000 cells/well (96-well plates for methylthiazolyldiphenyl-tetrazolium bromide [MTT] assay) in DMEM with 5% FBS, 100 U/mL penicillin, and 100 U/mL streptomycin in a humidified environment with 5% CO _2_ at 37 ℃ overnight. The cells were treated with CDDP (0, 1.25, 2.5, 5, and 10 μmol/L) for 24 h, and the treated cells were cultured in a 1% serum-reduced medium. RIF (10 μmol/L) was treated as a positive control.

### Primary mouse hepatocytes culture

Primary mouse hepatocytes were isolated from male ICR mice at the age of four weeks (obtained from the experimental animal center of Nanjing medical University, Nanjing, China), referred to the two-step perfusion method as was described previously with some modification ^[ 19] ^. The isolated primary mouse hepatocytes were plated at the density of 4 × 10 ^6^ cells/well into collagen coated 6-well plates and were maintained at 37 ℃, in a humidified atmosphere of 5% CO _2_ for 4 h to allow attachment. Primary mouse hepatocytes were then washed by PBS and supplied with fetal bovine serum-free medium. After being continually cultured for 2 days with a change of fresh medium, primary mouse hepatocytes were treated with CDDP (0, 1.25, 2.5, 5, and 10 µmol/L) for another 24 h. PB (1 mmol/L) was treated as a positive control.

### Animals and drug treatment

Thirty male ICR mice (4-week-old, 20–25 g weight, either sex) were obtained from Jiangsu Province's Medical Experimental Animal Center (Nanjing, Jiangsu, China). Mice were kept under environmentally controlled conditions (ambient temperature, 22 ℃; humidity, 40%) in a 12-h light/dark cycle with food and water *ad libitum*. After being domesticated for one week, mice were divided into three groups (control, CDDP-2.5, and CDDP-5, with 10 mice in each group). Mice in the CDDP-2.5 and CDDP-5 groups were injected intraperitoneally with CDDP 2.5 or 5 mg/(kg·day) for 3 days, respectively, and mice in the control group were injected intraperitoneally with the same volume of normal saline. The CDDP dosage in mice was converted from human dosage using body surface area normalization ^[ 20]^ and referred to the previous studies ^[ 21] ^. By the time of 24 h after the last injection for sacrifice, mice were intraperitoneally injected with ketamine (1 mL/kg at 100 mg/mL). When mice were completely anesthetized (approximately 5 min), surgery was performed to expose the livers. The liver was perfused with PBS through the portal vein to remove blood. The upper intestine of mice was isolated and washed with cold PBS. The perfused liver and washed intestine were then divided into two parts, with one part being immediately used for preparing total RNA and the other one frozen at −80 ℃ for preparing S9 fraction.

*Dec1* ^+/−^ C57BL/6 mice (RBRC04841) were obtained from the BRC (RIKEN BioResource Center, Japan). Heterozygous adult male mice ( *Dec1* ^+/−^) were crossed with adult female mice ( *Dec1* ^+/−^) to generate homozygous mice ( *Dec1* ^+/+^ and *Dec1* ^−/−^) ^[ 22] ^. Double checks (after birth and before the experiment) were applied to make sure the correct mouse genotype. The mouse genotype identification was presented in ***Supplementary Fig. 1*** (available online). Totally, 24 mice of two types ( *Dec1* ^+/+^, *Dec1* ^−/−^, *n* = 12 in each group, regardless of sex) were used. The total RNA and S9 fraction of the liver and intestine were prepared as described below.

The use of animals was approved by the Institutional Animal Care and Use Committee (IACUC, approval No. IACUC-2202043) of Nanjing Medical University. The procedures followed the guidelines for the care and use of animals established by the IACUC. Every effort was made to minimize animal suffering and to reduce the number of animals used for experiments.

### Preparation of S9 fractions

The frozen livers were thawed in homogenization buffer (50 mmol/L Tris-HCl, pH 7.4, 150 mmol/L KCl and 2 mmol/L EDTA), and then homogenized with a six-pass Teflon mortar and pestle driven by a Wharton stirrer. Homogenates were centrifuged at 10000 *g* at 4 ℃ for 20 min. After that, S9 fraction of the liver (supernatant) was assayed for the hydrolysis of PNPA and for Western blotting.

### Quantitative reverse transcription-PCR (qRT-PCR) assay

Total RNA was extracted by using TRIzol and checked by 1.5% agarose gel electrophoresis for quality control. The first-strand cDNA was synthesized by using the total RNA (1 μg) at 25 ℃ for 10 min, 42 ℃ for 50 min, and 70 ℃ for 10 min by using oligo (dT15) and M-MLV reverse transcriptase. qRT-PCR was performed by using FastStar Universal SYBR Green Master with the 7300 Real-time PCR System (Applied Biosystems, Foster City, CA, USA). The primers were shown in ***Table 1***. All data were normalized to the *GAPDH* or *ACTB*.

**Table 1:** The primers for human genes and mouse genes

Genes	Forward (5′→3′)	Reverse (5′→3′)
Human		
*CES1*	ACCCCTGAGGTTTACTCCACC	TGCACATAGGAGGGTACGAGG
*CES2*	CATGGCTTCCTTGTATGATGGT	CTCCAAAGTGGGCGATATTCTG
*PXR*	CGAGCTCCGCAGCATCA	TGTATGTCCTGGATGCGCA
*DEC1*	GGCGGGGAATAAAACGGAGCGA	CCTCACGGGCACAAGTCTGGAA
*CYP3A4*	TCAATAACAGTCTTTCCATTCCTCAT	CTTCGAGGCGACTTTCTTTCA
*GAPDH*	GTATGTCGTGGAGTCTACTGGTGTC	GGTGCAGGATGCATTGCTGACATTC
Mouse		
*Ces1d*	GGCATCAACAAGCAAGAGTTTGGC	CTTTTTGGTGAGGTGATCTGTCCC
*Ces1e*	TTCAAGGATGTCAGACCACC	AACACATTTTTTTTGATACAGGGTA
*Pxr*	GATGGAGGTCTTCAAATCTGCC	GGCCCTTCTGAAAAACCCCT
*Stra13*	ACGGAGACCTGTCAGGGATG	GGCAGTTTGTAAGTTTCCTTGC
*Cyp3a11*	CTTTCCTTCACCCTGCATTCC	CTTTCCTTCACCCTGCATTCC
*Actb*	TAAAGACCTCTATGCCAACACAGT	CACGATGGAGGGGCCGGACTCATC

### Enzyme activity assay

The total hydrolytic activity was spectrophotometrically determined with standard substrate PNPA. The treated cell suspension was sonicated by a sonifier (Nanjing, China), and the cell debris was precipitated by centrifugation at 12 000 *g* at 4 ℃ for 15 min. The supernatants or S9 fractions of mouse liver were assayed for hydrolytic activity toward PNPA as described previously ^[ 19] ^. A sample cuvette (1 mL) contained 10 µg of cell lysates or S9 fractions from the liver diluted in 100 mmol/L potassium phosphate buffer (pH 7.4), and substrate (1 mmol/L) at room temperature. Reactions were initiated by adding PNPA (10 μL of 100 mmol/L stock in acetonitrile), and the hydrolytic rate was recorded from an increase in absorbance at 400 nm. The extinction coefficient (E400) was determined as 13 mmol/(L·cm). Several controls were conducted including incubation with no protein.

### Cell viability and morphology

Cell viability was detected by the MTT assay. HepG2 cells were seeded into 96-well plates at the density of 5000 cells per well overnight. Cells were treated with CDDP (0, 6.25, 12.5, 25, 50, and 100 μmol/L) for 24 h. The same volume of PBS was as the control. Then, 20 µL of 5 mg/mL MTT was added to the cells, and the cells were incubated at 37 ℃ for another 4 h. The culture medium was discarded, and 0.1 mL DMSO was used to dissolve the precipitate. The absorbance at 570 nm [ *D*(570 nm)] was measured using an Automated Microplated Reader ELx800 (BioTek, Winooski, VT, USA).

After treatment with or without CDDP (5 μmol/L) for 12 h, the cells were washed twice with DMEM and treated with oseltamivir (100 μmol/L), clopidogrel (100 μmol/L), or CPT11 (40 μmol/L), respectively, for another 24 h. The morphological changes were observed and images were taken under an inverted light microscope (Nikon, Japan, at 22 ℃). Subsequently, the cells were subjected to the MTT assay.

For the synergistic effect assay, HepG2 or SW480 cells were seeded into 96-well plates at a density of 5000 cells per well overnight. The cells were treated with CPT11 (0, 5, 10, 20, and 80 μmol/L) alone or together with CDDP (5 μmol/L) for 24 h; or the cells were treated with CDDP (5 μmol/L) for 2 h first, and then treated with CPT11 (0, 5, 10, 20, and 80 μmol/L) (including CDDP) for another 22 h. After that cell viability was determined by using MTT, IC _50_ of CPT11 was calculated. For overexpression or knockdown experiments, transfected cells (vector, sh- *DEC1*, OE- *DEC1*), (vector, si- *PXR*, OE- *PXR*), or (vector, OE- *DEC1* + si- *PXR*, sh- *DEC1* + OE- *PXR*) were seeded into 96-well plates at a density of 5000 cells per well overnight. The cells were initially treated with CDDP (5 μmol/L) for 2 h, followed by treatment with CPT11 (0, 5, 10, 20, and 80 μmol/L) (including CDDP) for another 22 h. After that cell viability was determined by using MTT, and the IC _50_ of CPT11 was calculated.

### Modulation of PXR or DEC1 expression by RNA interference and overexpression

For the RNA interference experiment, HepG2 or SW480 cells were plated in 6-well plates overnight at the density of 6 × 10 ^5^ cells/well in DMEM supplemented with 5% FBS. Transfection was conducted with GenJet (Ver Ⅱ). With the transfection mixtures containing 1 μg of si- *PXR* construct or an equal amount of corresponding vector, the transfected cells were maintained for 48 h. Alternatively, the cells were lentivirally transduced with the most effective virus strains (LV-sh *DEC1* or LV-Con). After 12 h of infection with LV-sh *DEC1* or LV-Con (multiplicity of infection of 20), the purified *DEC1* knockdown cells were obtained by continued screening with medium containing puromycin. The transfected or infected cells were treated with CDDP (5 μmol/L) or DMSO (0.1%, v/v) for 24 h. Subsequently, the cells were harvested, and the protein was extracted. The expressions of CES1, CES2, CYP3A4, and PXR was determined by Western blotting analysis.

For overexpression, HepG2 cells were transfected with 1 μg of *PXR* (or *DEC1*) construct or an equal amount of corresponding vector. After 24 h of incubation, the transfected cells were treated with the same treatment as mentioned above. Western blotting analysis was used to determine the expressions of CES1, CES2, CYP3A4, and PXR. The cell lysates (60 μg for knockdown, 10 μg for overexpression) were detected for the efficiency of knockdown and overexpression of PXR (or DEC1) by Western blotting analysis.

### Luciferase assay

DEC1 promoter reporters (1.3 kb and 1.1 kb) were donated by Dr. Yan from the University of Rhode Island. HepG2 cells were plated in 48-well plates in DMEM with 10% FBS at the density of 1 × 10 ^5^ cells per well. The transfection was conducted by GenJet DNA Vitro Transfection Reagent (Ver. Ⅱ). The transfection mixtures contained 50 ng of a reporter plasmid (pGL3-DEC1-1.3-Luc or pGL3-DEC1-1.1-Luc) along with 5 ng of pRL-TK. After incubation at 37 ℃ for 12 h, the transfected cells were treated with either CDDP (5 μmol/L) or the same volume of PBS for another 24 h. Cells were washed twice with PBS and then lysed by passive lysis buffer (1×; Promega). The collected cells were performed to freeze/thaw two cycles. The reporter enzyme activities were determined with the dual-luciferase reporter assay system. This system contained two substrates, the firefly luminescence and Renilla luminescence, which were used to determine the activities of two luciferases sequentially. The firefly luciferase activity, reflecting the reporter activity, was measured by mixing an aliquot of 10 μL lysates with Luciferase Assay Reagent Ⅱ (Promega). After that, the firefly luminescence was quenched, and the Renilla luminescence was activated simultaneously by injecting Stop & Glo reagent (Promega) to the sample tubes. The firefly luminescence signal intensity was normalized based on the intensity of Renilla luminescence signal as normalized luciferase activity. The ratio of CDDP and PBS treatment normalized luciferase activity representing relative luciferase activity.

### Western blotting analysis

Cell (HepG2 or primary mouse hepatocytes) lysates (80, 40, 20, or 10 μg) or S9 fractions of mouse liver or intestine (30 μg) were resolved by 10% SDS-PAGE and electrophoretically transferred to a polyvinylidene fluoride membrane pretreated with methanol. After non-specific binding sites were blocked with 5% non-fat milk, the blots were incubated with an antibody against CES1 (1∶2000), CES2 (1∶2000), CYP3A4 (1∶2000), CYP3A11 (1∶2000), DEC1 (1∶2000), human PXR (1∶1000), mouse PXR (1∶1000), or β-actin (1∶5000). The primary antibodies were subsequently localized with goat anti-rabbit IgG conjugated with horseradish peroxidase. Horseradish peroxidase activity was detected with a chemiluminescent kit (Pierce). The protein bands were visualized with an enhanced chemiluminescence detection system. To eliminate the systematic errors between the sample adding and the different gels, the target proteins and the internal reference protein ran out on the same membrane. Therefore, membranes were horizontally stripped or cut and then reprobed. For example, samples were loaded on the same gel with a marker for detecting CYP3A4, CES1, and CES2. Then, they were electrophoretically transferred to a PVDF membrane. The membrane was horizontally cut along with 50 kDa into two parts. One membrane (> 50 kDa) was detected for CYP3A4, and then it was washed three times with TBS and stripped with Restor PLUS Western Blot Stripping Buffer for 15 min at 37 ℃ with gentle shaking to remove the blot. After being washed three times with TBS, the membrane (> 50 kDa) was detected for CES1 and CES2 successively with Western blotting. The other membrane (< 50 kDa) was detected for β-actin. Protein levels were quantified by density analysis using Image Analysis software (NIH), and expressed as interest protein/β-actin. Protein concentrations were determined with the BCA protein assay based on albumin standard.

### Statistical analysis

Data were represented as means ± standard deviation. Statistical analysis for multiple comparisons was performed using SPSS software (SPSS, version 22, Chicago, IL, USA). The significance was determined by one-way or two-way analysis of variance, followed by Tukey's post hoc test, and the paired comparisons were analyzed by Student's *t*-test. The differences were considered statistically significant when *P* < 0.05.

## Results

### CDDP induced the expression and activities of CES1 and CES2 in HepG2 cells

**Figure 1:**
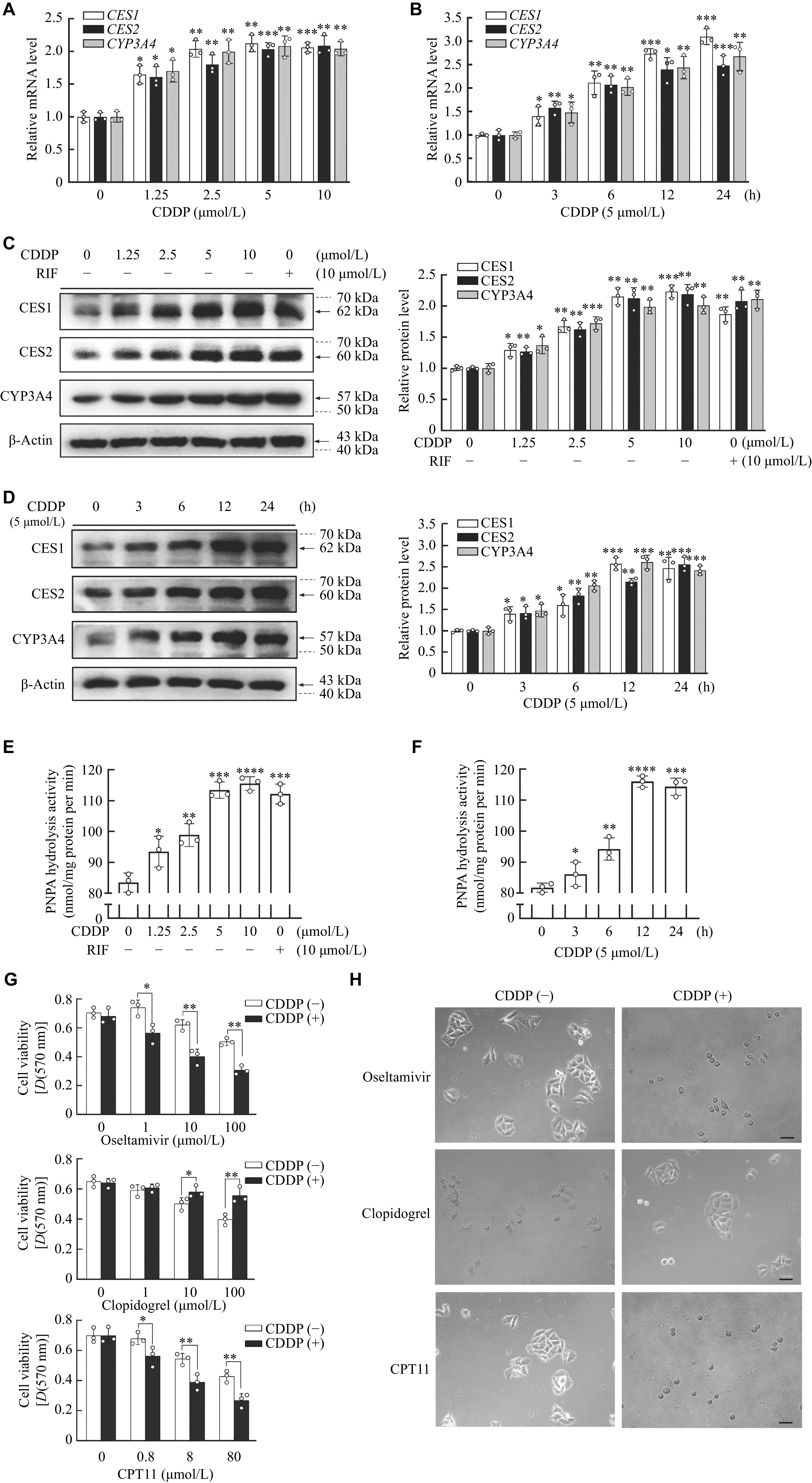
Induction of CES1 and CES2 expression as well as their activities in HepG2 cells.

First, we tested whether CDDP (6.25–100 μmol/L) affected the cell viability and determined the dose range of CDDP. As shown in ***Supplementary Fig. 2A*** (available online), when the dosage of CDDP exceeded 25 μmol/L, the cell viability dropped below 40% in HepG2 Cells. Therefore, the dose range of CDDP was set between 1.25 μmol/L and 10 μmol/L. HepG2 cells were treated with CDDP (1.25, 2.5, 5, and 10 μmol/L), or the same volume of PBS for 24 h or just one dose of CDDP (5 μmol/L, non-cytotoxic, ***Supplementary Fig. 2B ***, available online) for corresponding times (0, 3, 6, 12, and 24 h). As shown in ***Fig. 1***, the treatment with CDDP consistently increased both mRNA and protein levels of CES1, CES2, and CYP3A4 in a dose- and time-dependent manner ( ***Fig. 1A***– ***1D***). RIF also increased protein expressions of CYP3A4, CES1, and CES2 ( ***Fig. 1C***). Here, CYP3A4 acted as a positive control induced by CDDP ^[ 17] ^. Consistent with the increases in both mRNA and protein levels of CES1 and CES2, the overall hydrolysis of PNPA significantly increased both in a dose- and time-dependent manner ( ***Fig. 1E*** and ***1F***). It was noticed that the most significant induction of CDDP was at 5 μmol/L ( ***Fig 1A*** and ***1C***), whereas some cytotoxicity was detected in the cells treated with 12.5 μmol/L of CDDP, based on the MTT assay ( ***Supplementary Fig. 2A ***) and microscopic observation. Therefore, 5 μmol/L of CDDP (non-cytotoxic, ***Supplementary Fig. 2B ***) was chosen to investigate the mechanism in the following experiments.

Because PNPA was not a specific substrate for carboxylesterase isozymes, we subsequently investigated toxicological consequences of the increase of CES1 and CES2 induced by CDDP. According to previous studies, CES1 and CES2 differed markedly in the hydrolysis of oseltamivir (antiviral drug), clopidogrel (antiplatelet drug), and CPT11 (anticancer agent) ^[ 12, 23] ^. CES1 rapidly hydrolyzes oseltamivir and clopidogrel, whereas CES2 mainly hydrolyzes CPT11. Importantly, the hydrolysis of oseltamivir and clopidogrel is represented a higher or a lower toxicity than their parent drugs, respectively; while the hydrolysis of CPT11 is represented a higher toxicity than its parent drug. HepG2 cells were first treated with CDDP (5 μmol/L) for 12 h, washed twice with DMEM, and then treated with oseltamivir, clopidogrel or CPT11. After incubation for another 24 h, cell viability was measured by the MTT assay, and morphologic changes were detected under a microscope before the MTT assay. The cells pretreated with CDDP alone showed no difference in cell viability, compared with those non-pretreated with CDDP ( ***Fig. 1G***). However, cells pretreated with CDDP followed with oseltamivir (1, 10, and 100 μmol/L), clopidogrel (1, 10, and 100 μmol/L) or CPT11 (0.8, 8, and 80 μmol/L) presented statistically significant cell viability changes at the corresponding concentration, compared with those non-pretreated with CDDP ( ***Fig. 1G***). For example, the cells pretreated with CDDP exhibited a significant decrease in cell viability when exposed to oseltamivir at corresponding concentration, compared with those without CDDP ( ***Fig. 1G***, top). Oppositely, the cells pretreated with CDDP exhibited a significant increase in cell viability when exposed to clopidogrel at 10 μmol/L, compared with that non-exposed to CDDP ( ***Fig. 1G***, middle), implying that CDDP induced CES1 activity. In addition, the cells pretreated with CDDP exhibited a significant decrease in cell viability when exposed to CPT11, compared with that in non-pretreated cells ( ***Fig. 1G***, bottom), suggesting that CDDP increased CES2 activity. The changes in cell morphology were consisted with those in cell viability. As shown in ***Fig. 1H***, under a bright field, cells pretreated with CDDP were spread, and projects were well extended, while the cells without CDDP pretreatment were round, isolated, and aggregated when exposed to 100 μmol/L oseltamivir for 48 h ( ***Fig. 1H***, top). Conversely, cells pretreated with CDDP and exposed to 100 μmol/L clopidogrel for 48 h showed opposite results ( ***Fig. 1H***, middle). Likewise, when exposed to 80 μmol/L CPT11 for 48 h, cells without CDDP pretreatment were morphologically normal, whereas cells pretreated with CDDP were isolated, round and shrank ( ***Fig. 1H***, bottom). These data indicated that CDDP induced the CES1 and CES2 expression as well as their activities in HepG2 cells.

### Inverse regulation of PXR and DEC1 expression by CDDP in HepG2 cells

**Figure 2:**
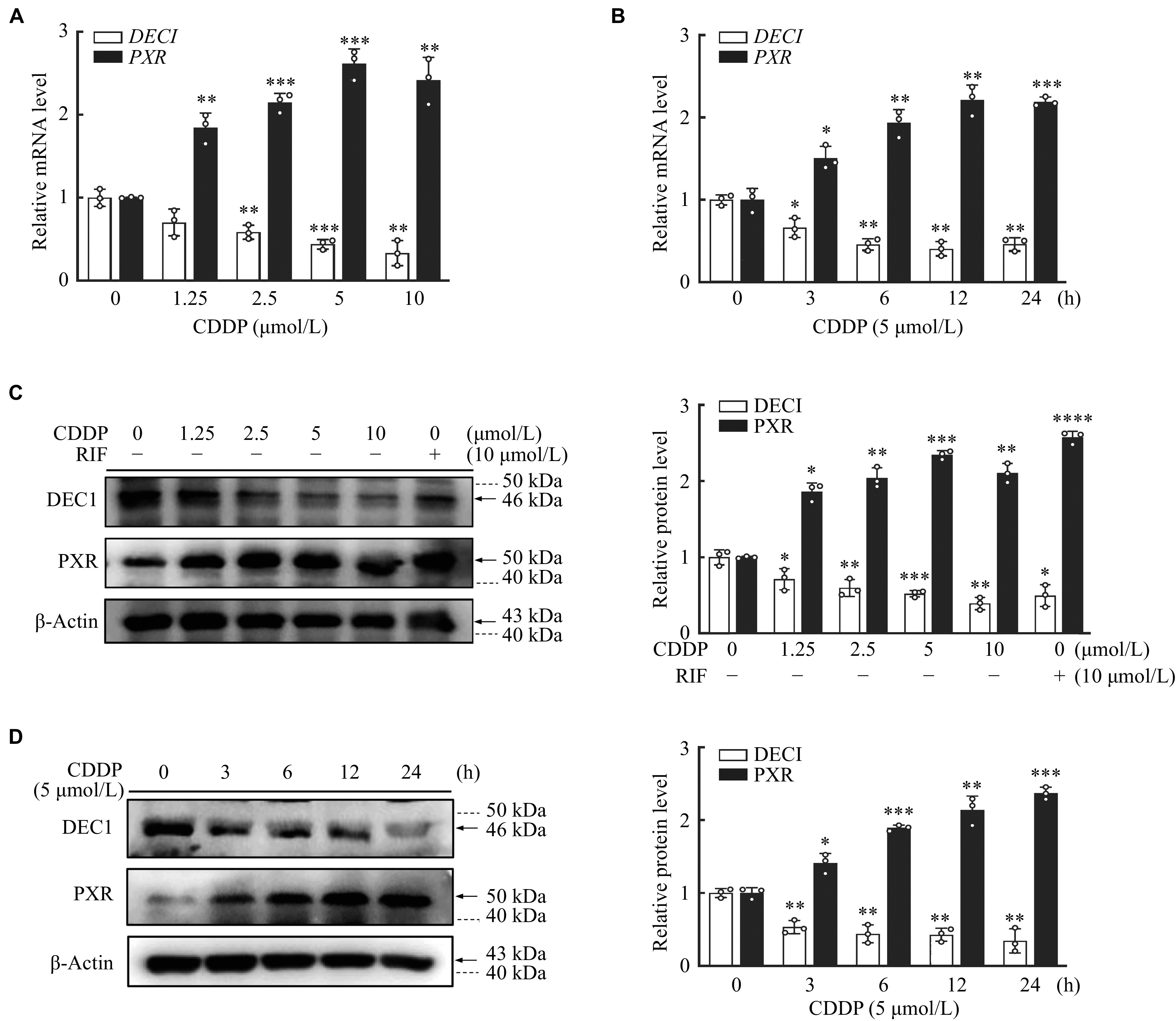
Inverse regulation of PXR and DEC1 expression by CDDP in HepG2 cells.

PXR is involved in the transcriptional regulation of CYP3A4 and CESs ^[ 24] ^, and DEC1 represses PXR by bounding to RXRα ^[ 17] ^. To explore the roles of PXR and DEC1 in the increase of CES1, CES2, and CYP3A4 expressions induced by CDDP, we examined the expressions of PXR and DEC1 in HepG2 cells treated with CDDP. As shown in ***Fig. 2***, CDDP stimulated PXR but inhibited DEC1 expression at both mRNA and protein levels in a dose- and time-dependent manner ( ***Fig. 2A***– ***2D***), suggesting that CDDP increased CES1, CES2, and CYP3A4 expressions along with increasing PXR but decreasing DEC1 expression.

To determine if primary mouse hepatocytes respond to CDDP similarly to human hepatoma cells in terms of the altered expression of carboxylesterases, primary mouse hepatocytes were treated with CDDP (0, 2.5, 5, 10, and 20 μmol/L) or phenobarbital (1 mmol/L, as a positive control), and cell lysates were prepared and analyzed for the hydrolysis of PNPA and the expression of carboxylesterases. Consistent with the results from HepG2 cells, the hydrolysis of PNPA markedly increased in primary mouse hepatocytes treated with CDDP in dose- and time-dependent manners ( ***Supplementary Fig. 3A*** and ***3B ***, available online). Likewise, the protein levels of CES1D and CES1E were comparably increased in dose- and time-dependent manners ( ***Supplementary Fig. 3C*** and ***3D ***). It should be noted that CES1D, and CES1E were detected by antibodies against human CES1 and CES2, respectively ^[ 25] ^. Consistent with that in human hepatoma cells, CDDP decreased STRA13 expression and increased PXR expression in dose- and time-dependent manners in primary mouse hepatocytes ( ***Supplementary Fig. 3C*** and ***3D ***). The data imply that the responses to CDDP in both human and mouse hepatocytes were similar.

### CDDP increased carboxylesterases expression and activities along with increased PXR and decreased STRA13 in the liver and intestine of mice

**Figure 3:**
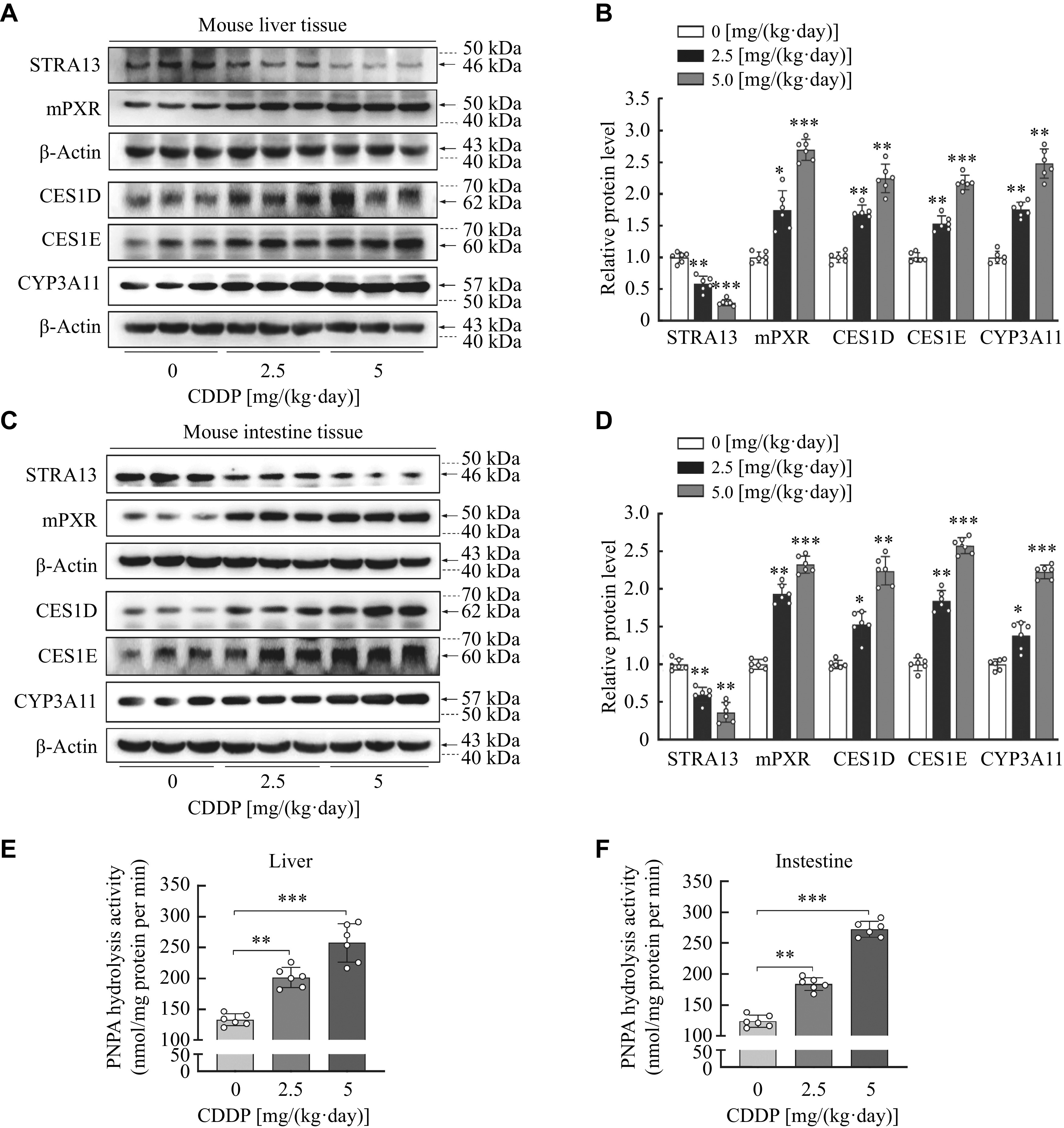
CDDP increased carboxylesterases expression and their activities along with increased PXR and decreased STRA13 in the liver and intestine of mice.

To confirm that CDDP can stimulate the carboxylesterases *in vivo*, mice were intraperitoneally injected with CDDP [0, 2.5 or 5 mg/(kg·day)], and S9 fractions of the liver and intestine were prepared and analyzed for carboxylesterases, *i.e.*, CYP3A11, PXR, STRA13 protein levels, as well as the hydrolysis activities. As shown in ***Fig. 3***, CDDP increased the expressions of CES1D, CES1E, and CYP3A11 in the liver and intestine in a dose-dependent manner, with an increase ranging from two-fold to three-fold ( ***Fig. 3A***– ***3D***). Likewise, comparable increases in hydrolysis activity of the liver and intestine induced by CDDP were detected in mice ( ***Fig. 3E*** and ***3F***). Consistent with that in HepG2 cells and primary mouse hepatocytes, CDDP decreased the STRA13 expression and increased mouse PXR expression ( ***Fig. 3A***– ***3D***). These results indicate that CDDP induced carboxylesterases expression and their activities along with the increased PXR but decreased DEC1 expressions *in vivo*.

### Involvement of PXR in the increase of carboxylesterases induced by CDDP

**Figure 4:**
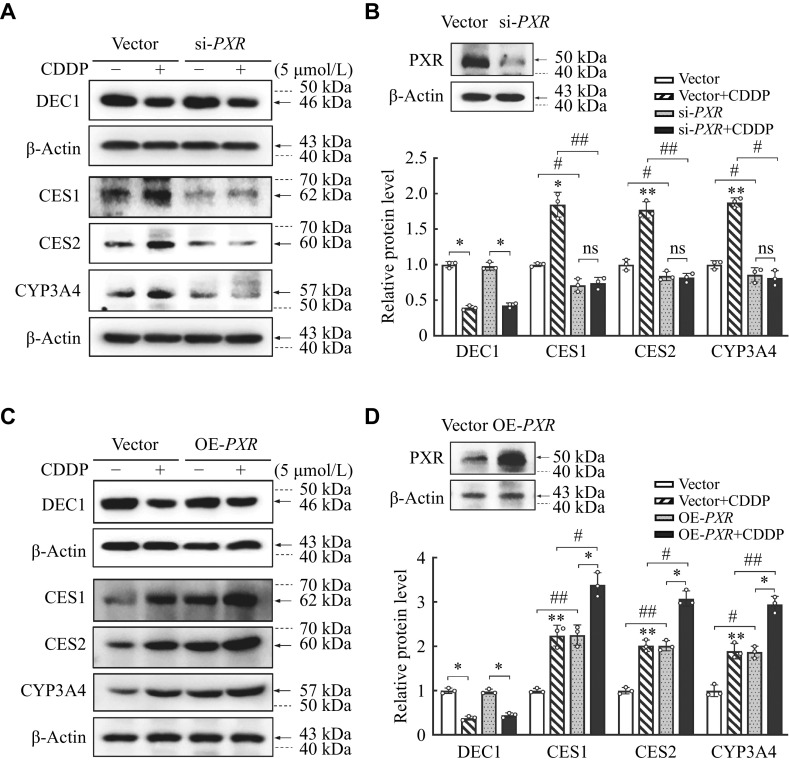
Involvement of PXR in the increase of carboxylesterases induced by CDDP in HepG2 cells.

Next, to test whether PXR plays a role in the increases of CES1, CES2, and CYP3A4 expressions induced by CDDP, we performed knockdown and overexpression of PXR experiments. As shown in ***Fig. 4***, CDDP increased the expressions of CES1, CES2, and CYP3A4 significantly in the vector group; whereas knockdown of *PXR* almost abolished these increases induced by CDDP with the knockdown *PXR* efficiency of more than 80% ( ***Fig. 4A*** and ***4B***). Conversely, the overexpression of PXR alone increased the expressions of CES1, CES2, and CYP3A4 significantly, compared with that in the transfected vector; moreover, it increased the expressions of CES1, CES2, and CYP3A4 much more than that in the transfected vector induced by CDDP ( ***Fig. 4C*** and ***4D***). It should be noted that neither the knockdown nor the overexpression of PXR changed the expression of DEC1 and the response to CDDP ( ***Fig. 4A***– ***4D***). These data imply that PXR was involved in the increase of carboxylesterases induced by CDDP, but not regulated DEC1 expression.

### Involvement of DEC1 in the induction of PXR and its targets by CDDP

**Figure 5:**
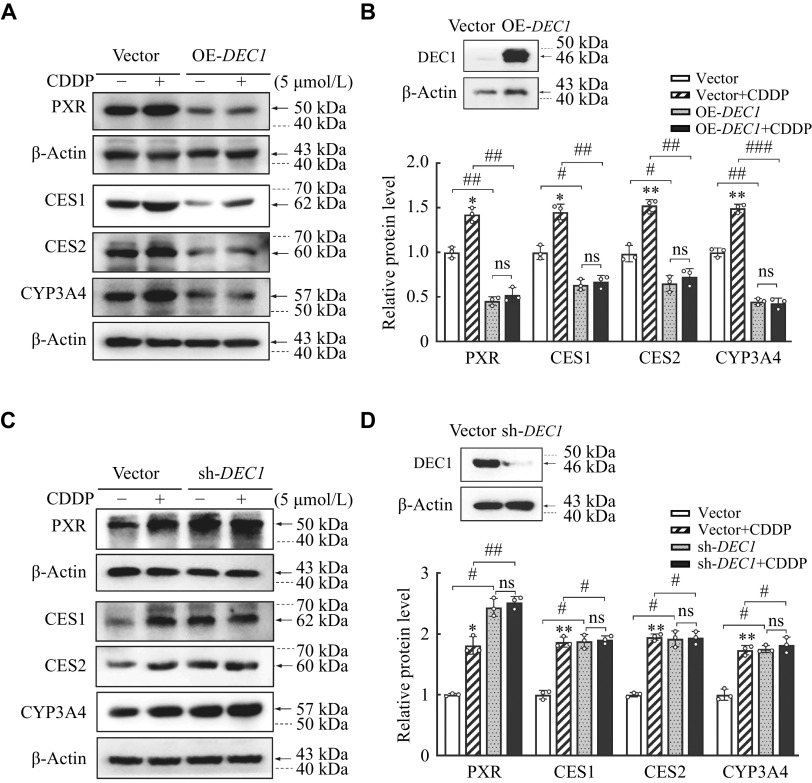
Involvement of DEC1 in the induction of PXR and its targets by CDDP in HepG2 cells.

Next, to test the role of DEC1 in the PXR and target genes induced by CDDP, we did the overexpression and knockdown of *DEC1* experiments. As shown in ***Fig. 5***, the overexpression of DEC1 alone decreased the expressions of PXR and targets, such as CES1, CES2, and CYP3A4, significantly, compared with that in the transfected vector; whereas the overexpression of DEC1 almost abolished the increased PXR and target expression induced by CDDP in the vector ( ***Fig. 5A*** and ***5B***). Oppositely, the knockdown of *DEC1* alone increased the expressions of PXR and targets CES1, CES2, and CYP3A4 significantly, compared with that in the transfected vector; however, the knockdown of DEC1 abolished these increases, compared with that in the transfected vector induced by CDDP ( ***Fig. 5C*** and ***Fig. 5D***). These results suggested that CDDP increased CES1, CES2, and CYP4A4 through activating PXR mediated by decreasing DEC1 expression.

**Figure 6:**
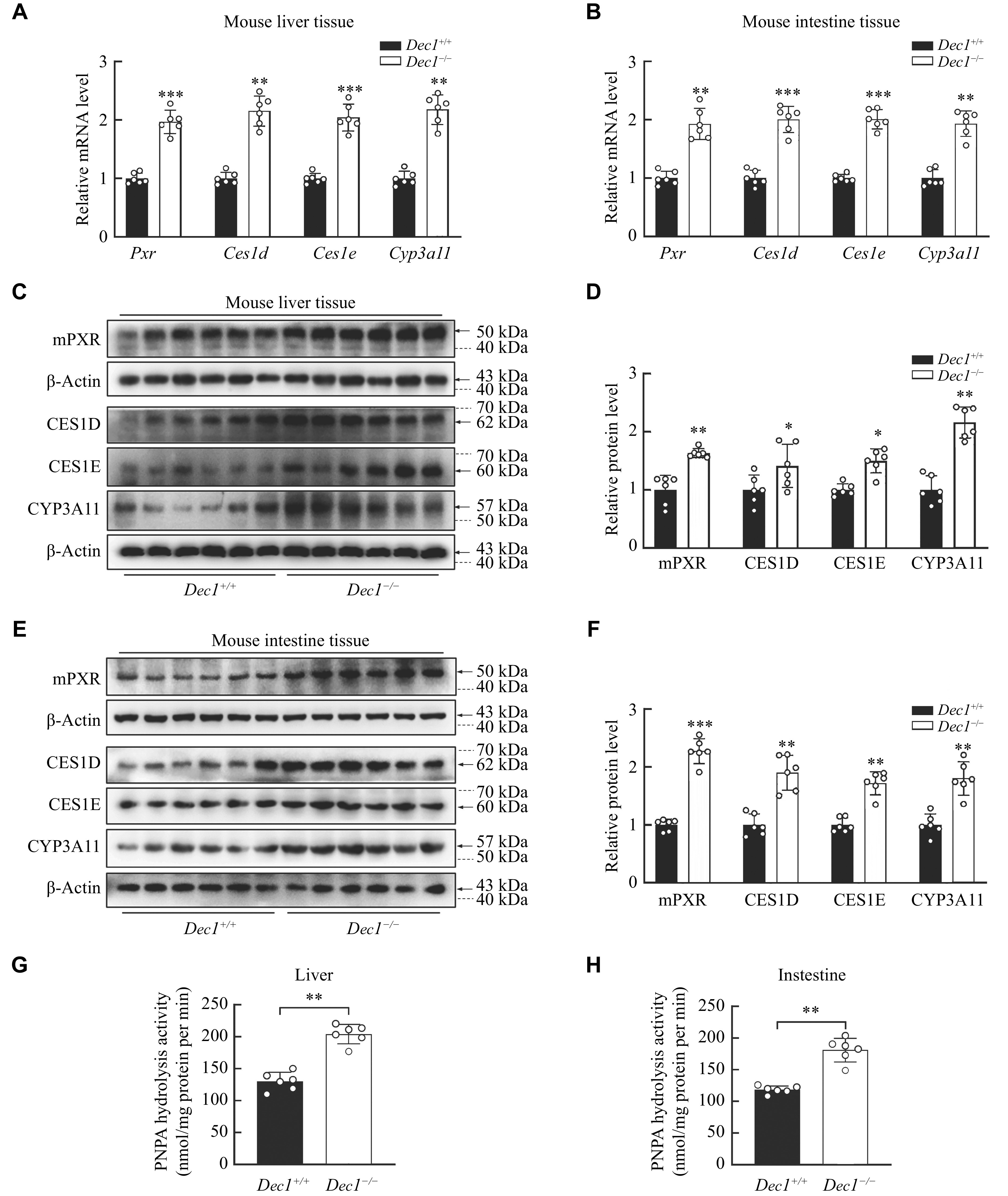
*Dec1* deficiency showed an increase of PXR and its targets in the liver and intestine of mice.

To validate the PXR and its target involvement of DEC1 *in vivo*, we examined the PXR and its targets, such as CES1, CES2 and CYP3A11, in the liver and intestine of the *Dec1* ^−/−^ and *Dec1* ^+/+^ mice (24 weeks old). Compared with *Dec1* ^+/+^ mice, *Dec1* ^−/−^ mice had the increased expression of PXR and its targets, such as CES1D, CES1E, and CYP3A11 at both mRNA ( ***Fig. 6A*** and ***6B***) and protein levels in the liver ( ***Fig. 6C*** and ***6D***) and intestine ( ***Fig. 6E*** and ***6F***) as well as the overall hydrolysis activity ( ***Fig. 6G*** and ***6H***). The increases of PXR and its targets were more than two-fold in *Dec1* ^−/−^ mice, compared with those in *Dec1* ^+/+^ mice, indicating that DEC1 was involved in the regulation of PXR and its targets *in vivo*.

### Transcriptional involvement in DEC1 suppression by CDDP

**Figure 7:**
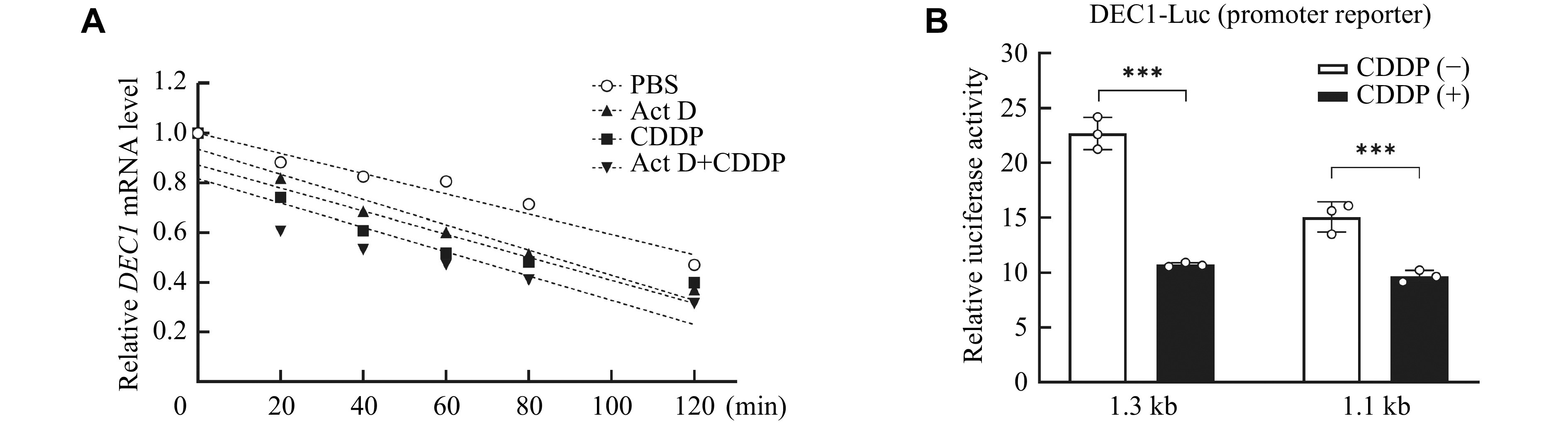
CDDP suppressed DEC1 transcriptionally.

The decrease of *DEC1* mRNA by CDDP suggests two possibilities: (1) CDDP suppresses the transcription and/or (2) increases the degradation of mRNA. To test the first possibility, a transcriptional inhibition assay was performed with CDDP in the presence or absence of the transcription inhibitor Actinomycin D (Act D). HepG2 cells were treated with CDDP (5 μmol/L) alone or together with Act D (2 μmol/L) for 0, 20, 40, 60, 80, 100, and 120 min. The total RNA was isolated and analyzed for the *DEC1* mRNA level. As shown in ***Fig. 7***, the slopes of the *DEC1* mRNA attenuated curve did not change when either being treated with CDDP alone or together with Act D ( ***Fig. 7A***), suggesting that CDDP decreased DEC1 expression not through the increase of degradation but through transcription suppression. This possibility was also tested with DEC1 promoter reporters. The DEC1 reporters contained 1.6-kb and 1.1-kb upstream sequences, respectively (kindly provided by Dr. Yan's Lab). HepG2 cells were transfected with a promoter reporter (DEC1-1.3-kb-Luc or DEC1-1.1-kb-Luc) and the Renilla plasmid, and the transfected cells were treated with CDDP or PBS. After a 48-h incubation, cells were lysed and the luciferase activities were determined. As shown in ***Fig. 7B***, treatment of CDDP significantly decreased the activity of DEC1 reporters. The transcription inhibition of DEC1-1.3-kb-Luc was 55%, which was comparable to the mRNA level detected by qRT-PCR ( ***Fig. 2A*** and ***2B***). Likewise, the transcription inhibition of DEC1-1.1-kb-Luc (33%) was less than that in the mRNA level. These data suggested the transcriptional involvement in DEC1 inhibition by CDDP.

### Enhanced cytotoxicity effect of CPT11 by CDDP through increasing PXR expression mediated by inhibiting DEC1 expression

**Figure 8:**
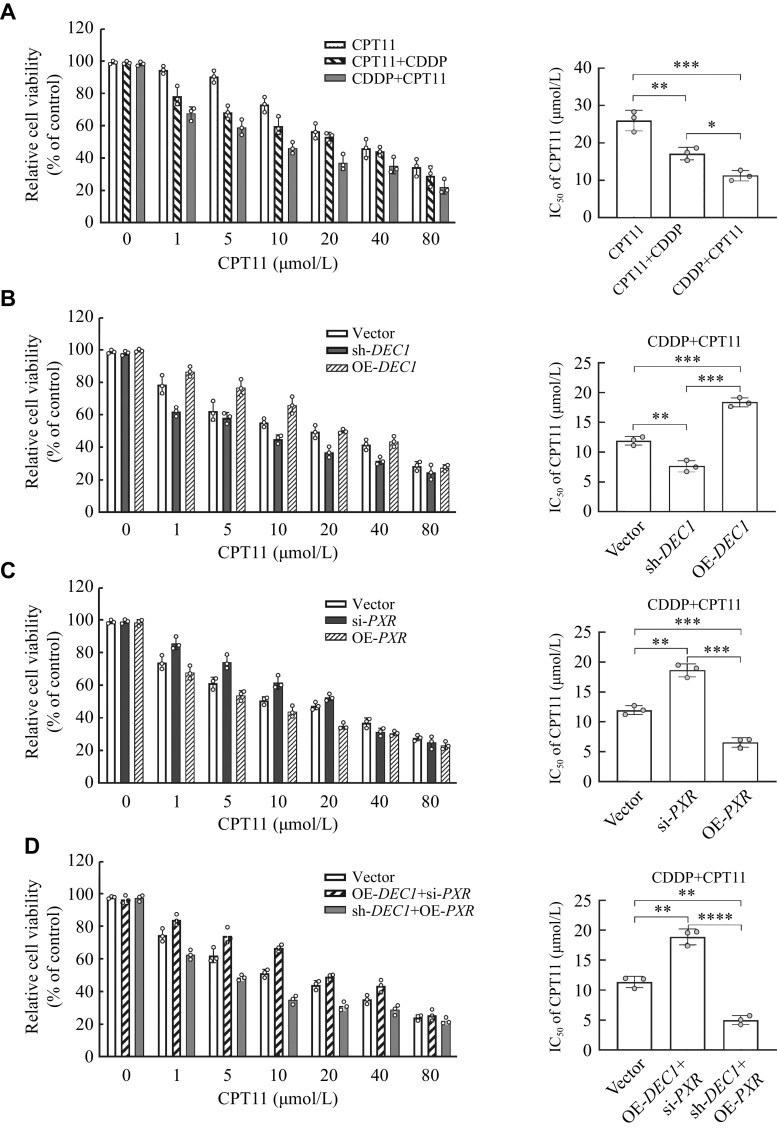
Enhanced effect of CPT11 by CDDP through increasing PXR expression mediated by inhibiting DEC1 expression in HepG2 cells.

To explore whether CDDP and CPT11 have a synergistic effect, HepG2 cells were seeded in 96-well plates at a density of 5000 cells/well overnight and treated with CPT11 (0, 1, 5, 10, 20, 40, and 80 μmol/L) alone, together with CDDP (5 μmol/L) for 24 h, or treated with CDDP (5 μmol/L) for 2 h first, and then added CPT11 (0, 1, 5, 10, 20, 40, and 80 μmol/L) for 22 h. The cell viability was determined by MTT. As shown in ***Fig. 8A***, IC _50_ of CPT11 alone, and together with CDDP (5 μmol/L) were 25.99 (± 2.73) μmol/L and 17.15 (± 1.66) μmol/L, respectively. Whereas, IC _50_ of CPT11 being treated with CDDP first and then added CPT11 was 11.26 (± 1.37) μmol/L ( ***Fig. 8A***, right). These results imply the combination effect of CPT11 with CDDP, especially being treated with CDDP first, and then added CPT11. It was noted that CDDP (5 μmol/L) was no cytotoxicity ( ***Supplementary Fig. 2B ***).

Next, we inquired into the roles of DEC1 and PXR in the synergistic effect between CDDP and CPT11. The transfected cells (Vector, sh- *DEC1*, OE- *DEC1*), (Vector, si- *PXR*, OE- *PXR*), or (Vector, OE- *DEC1*+si- *PXR*, sh- *DEC1*+OE- *PXR*) were seeded in 96-well plates at a density of 5000 cells/well overnight and treated with CDDP (5 μmol/L) for 2 h first, then added CPT11 (0, 1, 5, 10, 20, 40, 80 μmol/L) for 22 h. The cell viability was determined by MTT. As shown in ***Fig. 8***, the knockdown or overexpression of *DEC1* could enhance (IC _50_ of CPT11 was from [11.92 ± 0.72] μmol/L to [7.64 ± 0.94] μmol/L), or alleviate (IC _50_ of CPT11 was from [11.92 ± 0.72] μmol/L to [18.64 ± 1.08] μmol/L) the synergistic effect of CPT11 and CDDP significantly ( ***Fig. 8B***, right). Likewise, the knockdown or overexpression of *PXR* could alleviate (IC _50_ of CPT11 was from [11.99 ± 0.74] μmol/L to [18.64 ± 1.08] μmol/L), or enhance (IC _50_ of CPT11 was from [11.99 ± 0.74] μmol/L to [6.54 ± 0.80] μmol/L) the synergistic effect of CPT11-CDDP significantly ( ***Fig. 8C***, right). The knockdown of *DEC1* plus the overexpression of *PXR* could not enhance the synergistic effect of CPT11 and CDDP significantly, compared with that in the knockdown of *DEC1* or with that in the overexpression of *PXR* alone ([5.01 ± 0.74] μmol/L *vs.* [7.64 ± 0.94] μmol/L or [6.54 ± 0.80] μmol/L) ( ***Fig. 8D***, right). Also, the knockdown of *PXR* plus the overexpression of *DEC1* could not decrease the synergistic effect of CPT11 and CDDP significantly, compared with that in the knockdown of *PXR* or with that in the overexpression of *DEC1* alone ([18.88 ± 1.32] μmol/L *vs.* [18.64 ± 1.08] μmol/L or [18.36 ± 0.77] μmol/L) ( ***Fig. 8D***, right). Actually, the knockdown efficiency of *PXR* and *DEC1* was more than 70% ( ***Supplementary Fig. 4A ***, available online) and their overexpression efficiency was over three-fold ( ***Supplementary Fig. 4B ***, available online). Likewise, we got similar results in SW480 cells, which are summarized in ***Supplementary Fig. 2C*** and ***2D ***, ***4C*** and ***4D ***, and ***5 ***. These data suggested that the combination of CDDP and CPT11 had a synergistic effect, especially, in the concurrent sequential use of CDDP and CPT11. DEC1 and PXR were involved in the synergistic effect of CDDP and CPT11.

## Discussion

CDDP, widely used to treat a multitude of human cancers, such as cancers of lung, ovarian, breast, bladder, testicule, and brain, either alone or in combination with other drugs ^[ 26] ^, exerts its anti-tumor activity by covalently binding to DNA-forming adducts and therefore by triggering apoptosis ^[ 27] ^. CDDP is also used in combination with other antitumor drugs, such as CPT11 ^[ 28]^ and doxorubicin ^[ 29] ^. Therefore, it is critically important to elucidate the effect of CDDP on the drug metabolic enzymes that influence the drug-drug interaction. In the present study, we, for the first time, report that CDDP is an efficacious inducer of carboxylesterases. In HepG2 cells and primary mouse hepatocytes, CDDP markedly increases the expressions of CES1 (CES1D) and CES2 (CES1E) and hydrolysis activity as well as CYP3A4 (CYP3A11). Along with these changes, CDDP increases PXR expression, a major xenobiotic nuclear receptor that regulates CYP3A4 and carboxylesterases ^[ 24, 30] ^, and decreases DEC1 expression. These *in vitro* data are confirmed by animal experiments ( *in vivo*).

That the increase of CES1, CES2, and CYP3A4 by CDDP are abolished or proportionally increased when the expression of PXR is knocked down or overexpressed, and that the knockdown or overexpression of PXR alone decreases or increases CES1, CES2, and CYP3A4 expressions, it is assumed that the increases of CES1, CES2, and CYP3A4 by CDDP are caused by the increase of PXR. These conclusions are supported by other studies ^[ 13– 14] ^. But it seems to be in contrast with the reported effects in the kidney, where CDDP inhibits PXR expression in humans and mice ^[ 31] ^. The reason is that the dose of CDDP in making acute kidney injury is too large [20 mg/(kg·day) *vs.* 2.5 or 5 mg/(kg·day)]. It should be noted that the knockdown or overexpression of PXR do not change the DEC1 expression and neither do they change the decrease of DEC1 by CDDP. Thus, DEC1 is not regulated by PXR.

Next, we found that the overexpression or knockdown of DEC1 alone decreased or increased PXR expression, and abolished or alleviated the increases of PXR and its targets by CDDP. Thus, DEC1 is upstream of PXR and regulates PXR and its targets. These *in vitro* data are supported by the fact that the increase of mPXR and its targets in the liver and intestine was over twofold in *Dec1* ^−/−^ mice, compared with those in *Dec1* ^+/+^ mice, indicating that DEC1 was involved in the regulation of PXR and its targets *in vivo*.

The present study has provided some evidence to support that CDDP suppresses DEC1 transcriptionally. (1) The slopes of *DEC1* mRNA attenuated curve do not change either when treated with CDDP alone or together with Act D, implying that CDDP does not influence the degradation of *DEC1* mRNA and (2) CDDP significantly decreased the activity of DEC1 promoter reporters. Taken together, CDDP increases the expressions of CES1, CES2 and CYP3A4 through increasing PXR expression mediated by suppressing DEC1 transcriptionally. These data are consistent with the results of the decrease of PXR by IL-6 ^[ 17]^ and fluoxetine ^[ 30]^ mediated by the increase of DEC1. Thus, exogenous or endogenous substances (chemicals or cytokines) which influenced the expression of DEC1 affect PXR and target genes. Noda K *et al* have reported that CPT11 plus CDDP is an effective treatment for metastatic small-cell lung cancer, in a phase Ⅱ clinical trial ^[ 28] ^. Increasing clinical studies show that CPT11 plus CDDP is commonly used as community standard regimens for many advanced cancers ^[ 32– 33] ^. Clinically, with the recommended concentration of CPT11 (350 mg/m ^2^), the maximum plasma concentration is 3150 ng/mL (5.37 μmol/L) ^[ 34] ^, which is comparable to the concentration of CPT11.

The present study, for the first time, provides pieces of evidence that CDDP increases the activity of carboxylesterases, including CES1 and CES2: (1) CDDP markedly increases the expressions of CES1 (CES1D) and CES2 (CES1E) in HepG2 cells, mouse hepatocytes and mouse liver; (2) CDDP markedly increases the overall activity of hydrolysis; and (3) CDDP can increase the toxicity of oseltamivir and decrease the toxicity of clopidogrel, suggesting that it increases CES1 activity, whereas CDDP can increase the toxicity of CPT11, suggesting that it increases CES2 activity. More importantly, the synergy chemotherapeutic action of CDDP plus CPT11 is because of CDDP increasing the expression and activity of CES2, and it is through this mechanism that CPT11 is hydrolyzed to produce the active SN-38. IC _50_ of CPT11 in combination with CDDP is significantly lower than that in the use of CPT11 alone in two cell lines. Interestingly, the synergistic effect of CDDP and CPT11 is closely related to the order of the two drugs. Using CDDP first and then CPT11 is more conducive to obtaining synergy than concurrently using both. The reason is probably that the more CES2 is induced, the more CPT11 (SN-38) is activated, and the more synergistic effect is obtained. Pharmacologically, the involvement of PXR in CDDP-mediated induction implies that this chemotherapeutic agent causes more extensive drug-drug interactions in the clinic.
